# Perceptions about chronic health conditions, multimorbidity and self-management practices in rural northeast South Africa: findings from a qualitative study

**DOI:** 10.1136/bmjopen-2024-098219

**Published:** 2025-04-03

**Authors:** Audry Dube, Chodziwadziwa Whiteson Kabudula, Belinda J Njiro, Edward Fottrell, F Xavier Gómez-Olivé, Alisha N Wade, Stephen Tollman, Rochelle Burgess, Nicola Joan Christofides

**Affiliations:** 1 SAMRC/Wits Rural Public Health and Health Transitions Research Unit (Agincourt), School of Public Health, Faculty of Health Sciences, University of the Witwatersrand, Johannesburg, South Africa; 2 UCL Institute for Global Health, University College London, London, UK; 3 Research in Metabolism and Endocrinology, Department of Internal Medicine, School of Clinical Medicine, University of the Witwatersrand, Johannesburg, South Africa; 4 Division of Endocrinology and Metabolism, Perelman School of Medicine, University of Pennsylvania, Philadelphia, Pennsylvania, USA; 5 Health and Society Division, School of Public Health, Faculty of Health Sciences, University of the Witwatersrand, Johannesburg, South Africa

**Keywords:** Chronic Disease, Multimorbidity, Self-Management

## Abstract

**Abstract:**

**Introduction:**

Chronic health conditions are the leading causes of morbidity and mortality worldwide, with a disproportionately high burden in low-income and middle-income countries. The burden arising from these conditions presents immense challenges to countries with dysfunctional public healthcare systems, such as South Africa. This necessitates patients to have a good understanding of the conditions and optimal self-management approaches. We explored patients’ understanding of chronic health conditions and self-management practices, including self-monitoring, in the rural South African community of Agincourt in the subdistrict of Bushbuckridge, Mpumalanga Province.

**Methods:**

We randomly selected patients receiving routine care for chronic health conditions in primary healthcare facilities who were linked to the Agincourt Health and Demographic Surveillance System to participate in focus group discussions. Six focus groups (three with men and three with women) were conducted, with 17 male and 19 female participants (n=35) living with different chronic health conditions. Data were collected using body mapping exercises and semistructured focus group discussions facilitated by two experienced qualitative research assistants. An inclusive thematic approach was used for analysis.

**Results:**

Participants identified most chronic health conditions and their progression. Participants expressed that some consequences of chronic health conditions were unavoidable and some were attributed to medications. Three themes emerged on the management of chronic health conditions: (1) individual-level management, where participants actively changed or managed lifestyle factors associated with the conditions; (2) clinic-level management and support, where participants believed that following instructions from healthcare providers facilitates better management of their condition(s); and (3) prevention and screening, to prevent disease progression and development of complications. Participants also highlighted the role of religion in the control of chronic disease risk factors and traditional treatments for uncommon conditions such as epilepsy. Costs associated with lifestyle changes and equipment to manage and monitor health were highlighted as barriers to self-management of chronic health conditions.

**Conclusions:**

Our findings contribute to emerging research on chronic health conditions and self-management approaches. Participants in our study demonstrated a good understanding of various chronic health conditions but lacked knowledge of self-management practices and faced barriers to self-management. There is a need for further studies on self-management of chronic health conditions, including self-monitoring among patients in rural sub-Saharan settings.

**STRENGTHS AND LIMITATIONS OF THIS STUDY**

- We delved into the perceptions of self-management and self-monitoring among individuals with chronic conditions, a topic rarely explored in rural African settings, particularly in our study population.
- The body mapping method used in this study complemented the findings of focus group discussions by adding participants’ imaginations and visual insights, with this method expanding on the usefulness of using non-verbal cues in qualitative research.
- Participants recruited from the Agincourt Health and Demographic Surveillance System were homogenous in ethnic background, which may limit the generalisability of the findings in individuals residing in dissimilar settings.
- The focus group discussion method may have limited some participants from providing key personal experiences and voicing their concerns freely due to lack of privacy.

## Introduction

 Chronic health conditions, such as diabetes, arthritis, heart disease and HIV/AIDS, share many similar attributes in their management.^1^,^3^ These include managing complex medication regimens, dietary and lifestyle adjustments, regular exercise and adherence to clinic appointments.^1 2 4^ Given the high burden of multimorbidity (defined as the presence of two or more chronic health conditions) on patients, providers and healthcare systems, management of chronic health conditions requires integration of chronic healthcare services with home-based or community-based care.^5^ Most importantly, self-management and strong social and community support networks are key to navigating the complexities associated with managing chronic health conditions.^6^

Self-management encompasses the actions taken by an individual to manage symptoms, treatment, emotions and lifestyle changes associated with living with a chronic health condition.^1^ It involves patients actively learning more about their condition(s) and participating in their healthcare decisions. One of the key features of effective self-management is self-monitoring. The latter includes patients measuring and monitoring their clinical parameters (such as weight, blood pressure, blood glucose and lung function), symptoms (such as fatigue, stress, mood and pain) and features of daily life (such as physical activity, dietary intake and sleep patterns), often through tools such as diaries or health-tracking apps. These actions foster greater awareness of symptoms and bodily sensations^1^ and enable patients to bring about the cognitive, behavioural and emotional responses necessary to maintain a satisfactory quality of life, including seeking care when needed.^27^,^9^

Self-monitoring of chronic health conditions, including multimorbidity, is a well-established practice in high-income countries. Several studies, including randomised controlled trials and meta-analyses, have demonstrated its effectiveness, particularly in improving glycaemic control.^10^,^12^ However, in sub-Saharan Africa, the utility of self-monitoring in the management of chronic health conditions and multimorbidity is an active area of research. In South Africa, home-based screening and monitoring of blood pressure and antiretroviral therapy adherence, along with medication management and counselling, have been shown to improve blood pressure control and viral suppression in patients with both HIV and hypertension.^13 14^

While self-monitoring as a key feature of self-management is effective in improving clinical outcomes, understanding patients’ experiences with these activities is crucial, yet oftentimes overlooked. Studies have demonstrated that lack of adequate support during self-management or self-monitoring could worsen the impact of chronic health conditions.^15^ Research further indicates that patients face many challenges in self-managing their health, often due to lack of education, self-efficacy and social support for self-monitoring.^16^ In some cases, the challenges are caused by external factors such as healthcare providers’ attitudes during patients’ clinic visits and insufficient reassurance and explanations regarding the cause and management of the disease.^17^ Systemic challenges, including medication availability and long waiting times at healthcare facilities, also hinder effective self-management of chronic health conditions.^15 16 18^

Although self-management empowers patients to take care of their health, elements of social networks remain critical, particularly in lower-income communities where extended families play vital roles in patients’ health. A recent study focusing on the concept of ‘ubuntu’ highlighted that extended families provide not only practical assistance but also financial and emotional support to patients with chronic health conditions in managing their own health.^19^ Additionally, personal attitudes and perceptions towards self-management also present many challenges. For example, in many low-income settings, grandmothers are often the carers, and they often place their own healthcare needs aside while taking care of their families, further complicating self-management efforts.^20^

Despite some studies that have been conducted, there is still more to be done, particularly among rural populations in sub-Saharan Africa. While lack of knowledge about chronic diseases has emerged as one of the main barriers to self-management, it is crucial to understand how patients combine disease-specific knowledge with individual self-monitoring practices. This study, conducted in a rural South African setting, explored patients’ understanding and perceptions of chronic health conditions and self-monitoring using body mapping exercises and their intersection in implementing self-management. We also explored community perceptions and practices related to self-management of chronic health conditions and multimorbidity. The findings from this study provide valuable insights for policymakers and programme implementors, offering a deeper understanding of community perspectives on chronic health conditions, the experience of living with multimorbidity and the strategies used for self-management.

## Methods

### Study setting

This study was conducted within the Agincourt Health and Demographic Surveillance System (HDSS) study area in Bushbuckridge, Mpumalanga Province.^21^ The study area consists of 31 villages spanning approximately 450 km^2^ of semiarid land, with a total population of approximately 116 000 people. The population is served by a network of seven primary healthcare facilities located within the study area and two district hospitals located between 25 km and 40 km outside the study area. Although the Agincourt HDSS has noted an improvement in the socioeconomic status of people in the study area based on household assets, the area is still characterised by high levels of poverty and unemployment.^22^ Studies from the area also indicate that at least 60% of the adult population are currently living with one or more chronic health conditions.^14 23^

### Study design

This is a cross-sectional, formative qualitative study using focus group discussions (FGDs) and body mapping, with data collection done in May 2021. The study was part of a larger sequential mixed-methods study designed to assess knowledge, willingness and approaches to self-management among people with chronic health conditions. The findings from this formative research informed the development of quantitative questionnaires, which were administered to individuals with chronic health conditions who attended primary healthcare facilities in the Agincourt HDSS study area. FGD was chosen as the method of data collection due to its ability to facilitate interactive discussions that gather indepth information about complex experiences as well as the reasoning behind beliefs, perceptions and attitudes.^24^

### Study participants and sampling

The sample for the study included older adults diagnosed with at least one chronic health condition randomly selected from the Agincourt HDSS-Clinic-Hospital Link System database. The Agincourt HDSS-Clinic Link System is an ongoing longitudinal data platform that captures and stores clinical information collected from all primary healthcare facilities in the Agincourt HDSS study area and nearby district hospitals. Clinical information collected in the database includes symptoms at presentation, HIV testing and counselling, dates of collection of laboratory samples and test results (eg, CD4+ cell count and HIV-1 viral load), follow-up and clinic visits, vital signs at follow-up visits, and medications given.

### Data collection

Research assistants contacted and introduced the study to the selected participants by phone. Those who were interested in participating were invited to take part in the FGD on a set date. All FGDs and body mapping were completed at the field research facilities of the South Africa Medical Research Council (SAMRC)/Wits Rural Public Health and Health Transitions Research Unit. On the scheduled days, research assistants explained the aims of the study to the participants before starting each FGD.

Focus groups were formed, ranging in size from four to seven participants per group. While participants were initially recruited based on shared characteristics, such as age, the first focus group revealed that some participants in the older age groups had some hesitation or reluctance to express their opinions. Therefore, participants selected for subsequent FGDs had a mix of ages, which resulted in greater engagement from all participants. Mixing participants of different ages also made it feasible for the younger participants to assist in drawing and writing during the body mapping activities. Participants were grouped by sex to ensure comfortable participation and open discussion.

### Body mapping activity

We employed a body mapping approach to gain insight into the participants’ understanding of the impact of chronic health conditions on their bodies. This method allows participants to express their thoughts, sensations, emotions and physical pain through illustrated body representations, providing a comprehensive understanding of their health experiences and environment.^25 26^ Working in groups, participants identified chronic health conditions, their bodily manifestations and environmental factors that exacerbated or alleviated them using colours and symbols to represent each health condition. Participants were encouraged to focus on preventable or treatable chronic health conditions. Body maps were used as visual aids to illustrate anatomy and physiology, making it possible to understand people’s perceptions of their bodies and integrate the body, mind and social context.^27^

The body mapping exercise formed the first section of the discussion. Two trained research assistants facilitated the discussions. One assistant facilitated the discussion, while another captured notes from the observed non-verbal communication.

### Focus group discussions

A semistructured interview guide, presented in online supplemental file 1, was used to facilitate the FGDs. This provided a framework of open-ended questions or topics that guided the conversation while allowing for flexibility and spontaneity in the discussion. This allowed indepth exploration of topics and generation of rich insights from the participants while ensuring that the conversation stayed relevant to our research questions. The discussions were facilitated by trained research assistants who were familiar with the participants’ communities and culture. All FGDs were conducted in the local language, xiTsonga, and were digitally recorded and transcribed verbatim at a later stage. The research assistants then translated the transcripts into English before the analysis. Field notes were written after each FGD to reflect on the group and to capture initial impressions. These notes were not included in the analysis, but they helped improve subsequent FGDs.

### Data analysis

The data were analysed using Braun and Clarke’s six-step thematic analysis approach.^28^ The analysis used transcripts and illustrations from the body mapping exercise and the FGDs. A convergent approach was used to analyse the data from the body mapping exercise and the discussions. Key features illustrated in the drawings were further discussed during the FGDs.

Our analysis was driven by emerging themes identified in the data using an inductive (bottom-up) approach.^29^ Three researchers (RB, NJC and AD) conducted the data familiarisation step by reading and rereading the transcripts and generating initial codes. The codes were then reviewed and revised iteratively before the final set of codes was finalised. After reading most of the transcripts, the researchers met to review the themes, refined the themes and named all the themes before analysing the remaining data using the agreed approach. Themes were developed through observing patterns in the responses that reflected shared meaning. All emerging themes were documented during analysis, with agreement reached through discussions and consensus. We then selected key quotes for illustration under each identified theme. QDA Miner was used for coding and conducting the thematic analysis.

### Patient and public involvement

Patients or the public were not involved in the design, conduct or dissemination of our research.

### Ethical considerations

All participants provided written consent before participating in the study. The participants placed a signature or ink thumbprint on the consent form as proof of agreement to participate in the study. Participants were informed of their freedom to discontinue their participation at any point.

## Results

### Description of study participants

Table 1 shows the distribution of the participants in the six FGDs. A total of 35 participants (17 men and 18 women) took part in the study. Focus groups 1 and 2 were with the carers or members of the community taking care of a family member living with a chronic health condition. Most of the participants were living with hypertension and HIV/AIDS. Only two participants were living with diabetes, and three participants with other chronic health conditions mentioned having asthma, arthritis and ulcers.

**Table 1:** Characteristics of focus group participants

	FGD 1*	FGD 2*	FGD 3	FGD 4	FGD 5	FGD 6	Total
Participants, n	6	5	4	6	7	7	35
Mean age (SD)	52.57 (9.20)	60.20 (5.72)	53.00 (8.04)	55.00 (10.12)	58.43 (6.32)	57.86 (6.69)	55.7 (7.86)
Male to female ratio	4:2	2:3	4:0	0:6	7:0	0:7	17:18
Distribution of chronic conditions							
Hypertension	–	–	1	1	5	4	11
Diabetes	–	–	–	–	1	1	2
HIV	–	–	3	5	2	4	14
Asthma	–	–	–	1	–	–	1
Arthritis	–	–	–	–	1	–	1
Ulcers	–	–	–	1	–	–	1

* Focused group discussions with members of the community or carers.

FGDfocus group discussion

### Body mapping and community understanding of chronic health conditions

Participants understood chronic health conditions to include diseases that people live with for a long time, mostly for the rest of their lives. Commonly identified conditions included HIV/AIDS (all groups), hypertension (all groups) and diabetes. Some groups also identified conditions such as cancers, hearing impairment and epilepsy.

**Figure 1:**
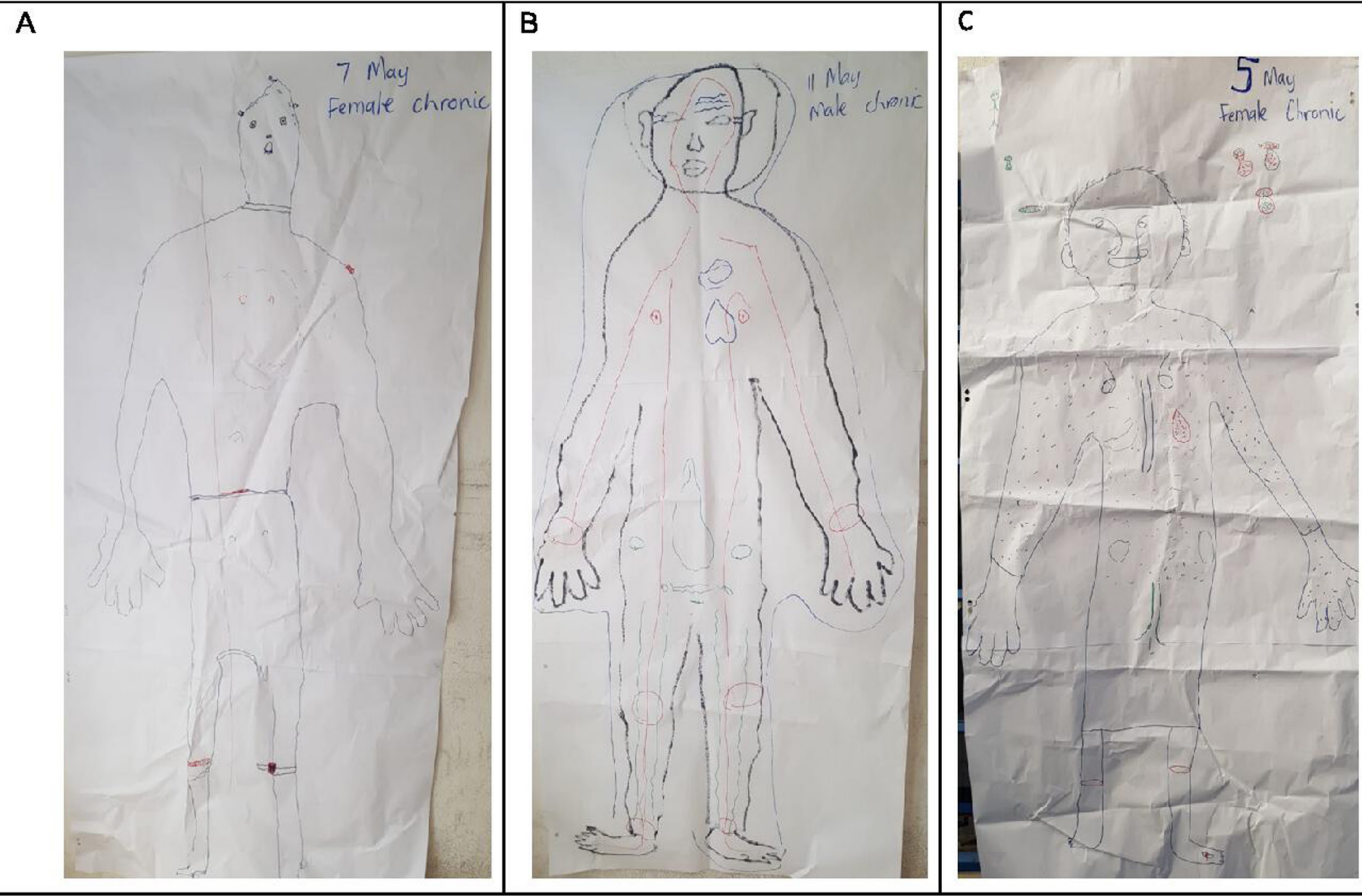
Body mapping drawings during focus group discussions. (A) An example of one of the body map drawings by the study participants. (B) Red circles on the wrists, knees and ankles indicate gout, a chronic disease that affects the joints. (C) Features images of salt, sugar and oil, which participants associated with increased hypertension.

In most cases, in the body mapping exercises, participants used different colours and symbols, such as dotted lines, to identify chronic health conditions, as illustrated in figure 1. Each drawing was unique, and different symbols were used to represent a chronic health condition. However, the ‘heart’ symbol was used consistently across all groups to denote hypertension.

We found that participants used different marks or symbols, such as a circle, a red dot or a line, on the leg or foot to indicate the sores that can develop as a consequence of poorly controlled diabetes. When asked about their drawings, most groups explained that people with diabetes developed sores that did not heal and this was a common consequence of diabetes (figure 1).

When someone has sugar diabetes, once they are scratched by something on their legs or anywhere in the body, they develop a wound that takes longer to heal. (P2, Female, FGD 2)

Another participant also used red circles on the wrists, knees and ankles to indicate gout, a chronic disease that affects the joints (figure 1B). In other groups, HIV/AIDS was denoted by dots or lines through the whole body to symbolise arteries and veins throughout the body. The development of lymph nodes behind the ears, pimples on the face and lighter lips were seen as signs of HIV/AIDS. Participants expressed that blood was responsible for the spread of disease in the body, while HIV/AIDS affected every part of the body.

In some cases, participants did not select the colours deliberately. Participants chose colours based on visibility or to differentiate between diseases. However, for two groups, the colour red was deliberately chosen to indicate danger, and in most cases HIV/AIDS was denoted by the colour red.

What made us choose the colour red is that we are saying those sicknesses are dangerous, so it stands for danger. (P8, Female, FGD 3)

Some participants added images outside of their body maps of family members whom they felt helped manage their conditions. The body maps also featured images of salt, sugar and oil, which participants associated with increased hypertension (figure 1C).

### Causes of chronic health conditions and multimorbidity

In some of the FGDs, participants mentioned that chronic health conditions can co-occur or act as risk factors for each other, and that certain conditions can exacerbate other conditions. For example, diabetes was perceived as responsible for worsening hypertension and mental illnesses.

Most of the people I know who have diabetes go to the clinic to get pills, and diabetes is a sibling to high blood pressure. Once you are angry, blood pressure rises and diabetes worsens, and one ends up having stroke and falling to death. (P6, Male, FGD 1)

Moreover, similar to hypertension, diabetes was deemed to be associated with high levels of stress. Participants believed that reducing stress and taking medications as prescribed could reduce the effects of diabetes. The participants also noted that inability to adhere to instructions by healthcare providers, such as adherence to medications or following a healthy diet, leads to more diseases.

As we go down, we passed from high blood pressure, sugar diabetes affects the heart and also causes other illnesses like ulcers hence we used two colours. It also affects the heart so much that it ends up not pumping well then, the ulcers get a chance to enter. (P8, Female, FGD 3)

Participants noted that emotions such as stress or sadness increased the risk of multimorbidity. During the FGDs, participants expressed that old age meant being reliant on their family members. Therefore, families not offering help causes stress, which could lead to high blood pressure and negative effects on diabetes.

Participants also believed that different chronic health conditions caused ‘diluted blood’ or were the result of ‘blood blockages’. These were seen as reasons why people developed other chronic health conditions. For example, participants noted that when there is virus in the blood, such as HIV/AIDS, other health conditions easily find their way into the body. Blood was seen as a vehicle that facilitated the development and spread of other diseases in the body.

The blood is the one that makes a person survive. On the issue of making a person survive, blood tends to let viruses get into it. When you are infected with HIV… it is in the blood, it is the blood that has let the HIV come in. Other things/diseases like diabetes and high blood pressure are related to the blood. The diseases dilute the blood. (P4, Male, FGD 2)

### Management of chronic health conditions

Participants’ understanding of self-monitoring was limited across all FGDs, with the term being unfamiliar to most of them. The concept that participants spoke about was related to taking care of themselves (self-management) in different ways, such as accepting their diagnosis as well as following instructions received from healthcare providers. Participants talked about activities that they engaged in to live well with their condition, which included taking their medications, exercising and eating healthy food.

What is needed for me to live well with my illness is to follow the doctor’s instructions. Never refuse a doctor’s advice, because what is failing us to live well with our illness and die soon is the failure to take medication according to the doctor’s prescription. (P1, Male, FGD 2)

Management of chronic conditions was linked to clinic-level management rather than self-management. Most participants relied on the clinic for health updates and explained that it was difficult to monitor their health completely on their own. Attending follow-up visits at the clinic and having vital signs measured, including blood pressure and blood tests, were considered essential in the management of chronic health conditions.

By doing tests time and again, tests in your body at clinics are the one that proves how you are in your body and it is what helps each and everyone but if we can try to avoid our tests and take maybe six months without going for tests it has too much danger somewhere because you might find yourself bedridden because of illnesses like diabetes and epilepsy, because you have taken so long without doing tests and you don’t know what is in your body, but if you go say, every three months we go the clinics and test and go along with the doctors we will be able to get help. (P4, Male, FGD 2)

Participants described their routine clinic visits and the tests done during these visits measure how well they were taking care of themselves. Blood pressure measurements or blood tests that were done at the clinic were used as an indicator of their health status. Moreover, participants noted that following instructions provided by healthcare providers is part of managing one’s health. Taking medications as prescribed and not missing clinic visits were viewed as ways of managing chronic health conditions. Although participants did not understand some language used by healthcare providers, they were happy to just be informed that they were doing well.

For high blood it is to take treatment in time that they have given to you and eat food that they have prescribed to you, and also wake up in the morning and do some exercise … do your household chores. (P3, Male, FGD 3)

I have to go to the clinic and ask them to take some blood samples so that I will check my well-being. (P5, Male, FGD 3)

Acceptance of one’s chronic health condition was also seen as critical to self-care. Participants felt that acceptance of living with chronic health conditions, even those more stigmatised, such as HIV/AIDS and epilepsy, meant that they would be able to cope with such conditions and manage their health. Accepting their conditions helped with managing fears associated with the disease, such as dying. Acceptance also led to the disclosure of their conditions to their loved ones and therefore likely reduced the worry and stress associated with having a chronic health condition. This also led to them having a supportive environment because their families were aware of their health conditions.

Is to accept that I have an illness. So basically, accepting that I am sick will help in taking care of myself. If I do not accept it myself who is going to accept me? I will end up not being fine. If I can understand myself and love myself then I can be fine, and I will be able to lead a better life going forward. (P1, Male, FGD 3)

To add, another thing is that you will be able to take your pills without any fear because if you keep it secret you will end up hiding those pills so that others do not see them, and if someone from the family finds the pills where you hide them and take them to other places because they do not know what the pills are for and you will also be fearful to ask about them. The disappearance of medication may make one miss treatment collection date, this may lead to default, and by so doing one ends up messing up with your own life. (P2, Female, FG2)

A supportive family was seen to aid in the management of chronic conditions. Family support helped participants with chronic health conditions, as families would remind them about taking their medications. Some participants thought family support was the reason why most patients with chronic health conditions were able to live longer. Support also extended to the community, where participants viewed this as a way in which they could discuss how they could work together to take care of those with chronic health conditions. Some participants noted that, although support groups were not common in their communities yet, they would be great tools for managing their health conditions.

When we are with our families right … we shouldn’t be scared to tell our wives about our illness, our wives are the ones who remind us most of the time and also to watch the treatment that we are taking, we take it accordingly, our wives are the ones that are helping the nurses … in other words, our wives are our nurses at our homes, and they are the ones who will always be in our lives, it is what is helping us to be always healthy at home…. (P6, Male, FGD 2)

When asked to elaborate further on what methods they use when managing their health conditions, participants talked about practising health behaviours, such as healthy eating, as advised by healthcare providers, by minimising or avoiding salt and sugar and increasing physical activity.

For us to live well with our illness, we have to take our treatment on time, eat healthy food, do some exercise, and all these teach good health behaviours to those we are living with and to the community. (P2, Male, FGD 2)

The role of cultural and religious beliefs in dealing with some chronic diseases, such as epilepsy, was also raised. Participants highlighted that some chronic diseases needed to be treated traditionally. Others reported religion to be helpful for them in terms of avoiding some of the risk factors for chronic diseases, such as abstaining from alcohol use and smoking.

I think epilepsy falls to mental health illness because it goes with falling and it is not curable also and you have to live with it for the rest of your life … but epilepsy is not that famous because they say it is something that needs to be treated traditionally and nowadays those traditional treatments are failing…. (P4, Male, FGD 2)

So what is good is that you should take treatment for this illness and don’t drink alcohol or smoke cigarettes. So I can say I’m also helped by religion because I don’t take in alcohol; it is only to go to the party and eat there…. (P3, Male, FGD 2)

Some participants felt that following healthy eating behaviours was challenging because they had to start eating different foods from their families. Others felt that running as a physical activity was a difficult challenge and something they would not be comfortable engaging in due to old age and arthritis.

### Barriers to self-management

From our focus groups, two participants reported self-monitoring their health using devices, such as blood pressure monitors and glucose metres, to manage their chronic health conditions.

I do have a high blood pressure machine. (P1, Male, FGD 3)

Interviewer: What do you do with your BP [blood pressure] machine?

I use the machine to check my blood pressure time and again. (P1, Male, FGD 3)

However, participants expressed that one of the challenges in self-monitoring their health conditions was the unaffordable prices of the self-monitoring instruments. Going to a clinic for monitoring was viewed as the more affordable option.

If you are financially stable, with things like sugar diabetes and high blood pressure, buy yourself instruments that are similar to those they use at the clinic… like a BP machine buy it and have it and you will be able to measure your BP at home and diabetes, you can test yourself while you are at home… so but those go according to your financial status and if you’re not financially stable continue by going to the clinic or a health center that is close to you. (P2, Male, FGD 5)

Food recommended by healthcare providers was also described as expensive. Participants expressed not being able to regularly follow the recommended diet due to financial constraints. While following healthcare providers’ recommendations is key to achieving the best health outcomes, failure to follow eating plans as instructed by healthcare providers was pointed out to cause stress.

Eh……the only change in the homes we are staying in especially because we come from different backgrounds the food we eat must change, it requires one to eat the food or follow the diet that the doctors have told you to eat. Food is expensive nowadays so if the doctors have told you to eat certain food and you found that it is impossible to get that kind of food, it does create stress and affects the body because once a person is stressed because they can’t get, what they are required to eat, it affects that person physically and mentally. They worry about where are they going to get the required food because they can’t afford to buy it. That is when you develop stress and then do not recover because of too much thinking. (P5, Female, FGD 3)

Participants noted that taking care of their health meant that they would live long. They felt that if they were able to self-manage their health, they would be able to reduce the burden of the disease in their bodies and make their families proud.

## Discussion

This study explored the understanding and perceptions of chronic health conditions, multimorbidity, self-management and self-monitoring among older people in a rural South African setting. We found that participants were aware of various chronic health conditions that were prevalent in the community, including HIV/AIDS, hypertension and diabetes. Beliefs that having one condition made a person susceptible to getting another condition were how people understood multimorbidity. Beliefs about diluted blood and blood blockages were used to explain how one condition led to another. Generally, the concept of self-monitoring was unfamiliar to participants. Visiting the clinic, taking medications and following the advice of nurses were considered important ways of managing chronic health conditions.

Most conditions, such as hypertension, diabetes and HIV/AIDS, were widely recognised, with participants able to identify their characteristics, causes and ways to manage them. Their understanding of chronic health conditions was shaped by prevalent representations of the diseases in their communities and the information provided by the healthcare providers.^30^ Participants were able to identify chronic health conditions, their symptoms and their effects on the body. Some conditions, like diabetes, were understood to be diagnosed based on complications such as ulcers, kidney failure and blindness. These were illustrated using body maps and story-telling. However, there was limited understanding of the term ‘self-monitoring’, but with some good understanding of the term ‘self-management’. Participants expressed their understanding of the concept by eating healthy, avoiding stress and adhering to their medications. Despite being exposed to these, participants did not regularly monitor different health parameters to better understand their health. These findings are similarly reported in other African settings, where understanding self-monitoring does not necessarily result in the motivation to engage.^16^ This calls for more robust self-monitoring models that are patient-driven and address the disconnect between facility-based services and patients’ lived experiences.^15^

It was also noted that understanding and self-management of some conditions were entrenched in cultural and religious beliefs. This was the case especially when uncommon conditions such as epilepsy were discussed. These findings showed that, at a fundamental level, cultural and religious beliefs inform individuals’ health, their knowledge of health conditions, their experiences and the actions that they take to live with chronic health conditions.^30 31^ Education and raising awareness may not be sufficient to effect sustainable behavioural change for some individuals, as moral conflicts can arise between individualistic and collectivist goals.^32^ Religion was seen as having a positive influence towards avoiding some risk behaviours for chronic diseases, such as abstaining from alcohol use and cigarette smoking. This has been demonstrated elsewhere, where religious beliefs have been demonstrated to promote behavioural changes that reduce the burden of chronic non-communicable diseases.^33 34^

One crucial element of self-management is ensuring that patients prioritise their health by focusing on all the health conditions that they may have. However, among our participants, there was a focus on conditions they were most afraid of, such as diabetes and HIV/AIDS. Previous evidence has also identified fear of complications as a key factor that influences self-management of chronic diseases and healthcare utilisation behaviours, including clinic attendance.^35^ In our study, we noted that among participants with multimorbidity, proper self-management involved adherence to the prescribed instructions for that one chronic health condition that they feared most. In some studies, fear of negative health outcomes was associated with avoidance behaviours, such as avoiding medical appointments or necessary tests, which can worsen the patient’s condition.^36^

There was an emphasis on what people living with chronic health conditions did to enable them to live healthy lives. This included learning skills related to managing and coping with chronic health conditions. Similarly, coping included being open about their condition to family members and transitioning to living with the condition. This meant that participants were able to ask for assistance when needed. Moreover, they expressed that when families knew about their conditions, such as hypertension, the families would be able to accommodate their needs, such as avoiding stressful situations. This finding emphasised the importance of family when dealing with chronic health conditions. Similar evidence highlighted the crucial role of immediate family and relationships in assisting with medications and clinic visits, emotional support and daily care.^15^ The adjustments to living with chronic conditions must also involve the balance between healthy family support and avoiding putting too much demand on family caregivers.^18 37^ Another aspect of coping was associated with acceptance of their conditions. This was found to be necessary to learn to process the emotions associated with having a chronic health condition.

Healthcare providers were central to how participants managed their health conditions. Our study showed a reliance on healthcare providers and services to manage chronic health conditions. Regular check-ups and adherence to appointments were considered an important part of self-management practices for chronic health conditions. Patients relied on healthcare providers to give them a ‘green light’ on how well they managed their health, leaving patients ill-informed about what exactly good progress entails. We found that patients were glad to be told that they were doing well and understood that they had to continue following instructions from the healthcare providers. However, a lack of self-efficacy and autonomy in their health could hinder the implementation of self-management.^38^ Additionally, we observed from patients’ perspectives a lack of emphasis from healthcare providers on promoting and encouraging self-monitoring of patients’ health.

The financial implication associated with acquiring self-monitoring devices was one of the main barriers to self-monitoring in our setting. Participants, therefore, preferred to receive testing and monitoring for free in healthcare facilities. Similarly, the diets recommended by healthcare providers were considered to be expensive options that most individuals could not afford on a regular basis. These financial barriers have been documented in previous studies and span from patient-level to provider and health system factors, including patients’ inability to buy food and medications, stockouts and unavailability of monitoring instruments at the facilities.^17 36 39^

### Limitations

Body mapping was harder for older participants, especially those with conditions such as arthritis, which made drawing more difficult. Initially, older participants were hesitant and found it challenging to express themselves. This was mitigated by having a mix of ages in most of the groups. Although the FGDs were formed based on living with any chronic health condition, these mostly included hypertension, HIV/AIDS and diabetes, as these are the most prevalent chronic diseases in the Agincourt HDSS study area. This may have influenced which diseases were focused on during the FGDs, and our findings may not be generalisable to populations with other less prevalent chronic health conditions. Additionally, social desirability bias may have influenced participants’ responses, as they might have provided facilitators with answers they believed were expected.

## Conclusion

This study has explored the understanding of chronic health conditions and self-management practices within a rural South African community. Participants understood the common chronic conditions and illustrated the main complications associated with living with chronic health conditions. They also highlighted some reliance on religion in the prevention of risk factors for chronic diseases and the belief in traditional treatments for less common chronic diseases. The study found that self-monitoring was not a common practice among participants and that the concept of self-monitoring was not well understood. This is perhaps because the term is not often used in daily life and is mostly used in spheres of health policy and professional practice. However, we found that healthcare providers, and in some instances family members, assisted in the self-management of chronic health conditions. Patients heavily relied on healthcare providers for the management of their conditions, and this was exacerbated by limited financial resources to invest in the devices for self-management and self-monitoring.

The findings of this study provide opportunities to explore and develop self-monitoring tools and research initiatives to enable patients to self-manage their health for a longer and healthier life. Lastly, there is still a need for more information regarding self-monitoring of chronic conditions in these settings. The implementation of policies and programmes should be patient-driven to ensure that patients are appropriately informed and educated on self-monitoring of chronic health conditions, as this is an integral part of successful self-management practices.

## supplementary material

10.1136/bmjopen-2024-098219 online supplemental file 1

## Data Availability

Data are available upon reasonable request.
