# Supplementary material for: Perceptions about chronic health conditions, multimorbidity and self-management practices in rural northeast South Africa: findings from a qualitative study
Source: BMJ Open. 2025 Apr 3;15(4):e098219. doi: 10.1136/bmjopen-2024-098219 (PMC11969589; doi:10.1136/bmjopen-2024-098219)
Supplement: online supplemental file 1 [file bmjopen-15-4-s001.pdf]

Focus group discussion guide for self-monitoring: community

Start with body mapping – draw an outline of a body. Draw where the illnesses are that people living in this community have, make sure that you draw those illnesses that people have to live with for a long time, even the rest of their lives. How do they treat these problems or take care of them?

Awareness of symptoms/bodily sensations

1. What illnesses are common in the community? Think about illnesses that people live with for many years or even the rest of their lives?
2. How do people know or suspect that they have one of these illnesses?

Self-care

3. What are some of the things that people have to do to live well with their illness?

Measurement / observation / recording

4. How do people know that they are managing their illnesses well?
5. What are some of the things that people do to measure or observe how they are doing? (e.g. monitoring weight, exercise, medication, symptoms of depression)
6. Which of these things have you done?
7. Tell us about your experience of doing [activities mentioned under q.6]

Meaning of self-monitoring

8. What do people call this process of watching and recording things like weight, exercise, medication, blood pressure or sugar?
9. What does it mean to you look after yourself in this way?
